# Fibroblast Yap/Taz Signaling in Extracellular Matrix Homeostasis and Tissue Fibrosis

**DOI:** 10.3390/jcm13123358

**Published:** 2024-06-07

**Authors:** Cong-Qiu Chu, Taihao Quan

**Affiliations:** 1 Division of Arthritis and Rheumatic Diseases, Oregon Health & Science University, Portland, OR 97239, USA; chuc@ohsu.edu; 2 Rheumatology Section, VA Portland Health Care System, Portland, OR 97239, USA; 3 Department of Dermatology, University of Michigan Medical School, Ann Arbor, MI 48109, USA

**Keywords:** fibroblast, Yap/Taz, fibrosis, ECM, immune cell, mechanical force

## Abstract

Tissue fibrosis represents a complex pathological condition characterized by the excessive accumulation of collagenous extracellular matrix (ECM) components, resulting in impaired organ function. Fibroblasts are central to the fibrotic process and crucially involved in producing and depositing collagen-rich ECM. Apart from their primary function in ECM synthesis, fibroblasts engage in diverse activities such as inflammation and shaping the tissue microenvironment, which significantly influence cellular and tissue functions. This review explores the role of Yes-associated protein (Yap) and Transcriptional co-activator with PDZ-binding motif (Taz) in fibroblast signaling and their impact on tissue fibrosis. Gaining a comprehensive understanding of the intricate molecular mechanisms of Yap/Taz signaling in fibroblasts may reveal novel therapeutic targets for fibrotic diseases.

## 1. Introduction

Tissue response to disease and injury often leads to the accumulation of dense scar tissue, a phenomenon collectively referred to as fibrosis [1]. Fibrotic scarring is a widespread phenomenon affecting virtually every tissue in the body. It often emerges as a common pathological consequence of various chronic diseases [2,3]. Dysfunctional healing through fibrotic scarring can lead to structural abnormalities within organs. This leads to organ malfunction and lifelong disability, placing a substantial burden on public health [4]. Collectively, fibrosis is implicated in 35% of all deaths worldwide [5]. As such, fibrosis is increasingly recognized as a significant cause of morbidity and mortality in many chronic diseases. However, the lack of effective therapies is primarily due to a limited understanding of the underlying mechanisms that drive fibrosis development [4].

In adult mammals, when tissues or organs are damaged, they typically do not regenerate. Instead, a fibrotic scar forms, characterized by an excessive buildup of abnormally structured and tightly packed collagen fibrils, a hallmark of fibrotic scaring. The human skin, for example, heals wounds through fibrotic scars. Scars exhibit the following three key distinctions from normal skin [6]: (1) absence of dermal appendages, including hair follicles and sebaceous glands; (2) fibrotic collagen with dense and parallel bundles of fibrils; and (3) reduced flexibility and strength because of altered dermal collagenous extracellular matrix (ECM) structure. Although advancements have been made in reducing scarring, achieving scar-free wound healing remains elusive. As a result, the complete regeneration of skin with hair follicles and glands still poses a significant clinical challenge.

The intricate interplay between fibroblast signaling and ECM dynamics holds a critical role in the process of tissue fibrosis. Among the key orchestrators of this complex process are the Yes-associated protein (Yap) and Transcriptional co-activator with PDZ-binding motif (Taz) pathway [7,8]. Fibroblasts, traditionally recognized for their role in ECM synthesis and deposition, extend their influence beyond providing structural support [9]. They actively engage in various physio-pathological processes, such as inflammation and angiogenesis, contributing to the complex network of cellular events governing tissue fibrosis. This review focuses on current understanding about the involvement of Yap/Taz in fibroblast signaling, their role in normal ECM maintenance, and their dysregulation leading to tissue fibrosis.

## 2. Role of Fibroblasts in Tissue Fibrosis

**Figure 1:**
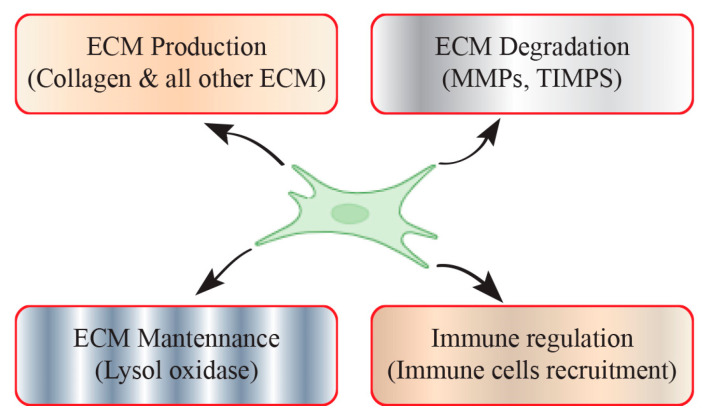
Fibroblasts play a pivotal role in maintaining ECM homeostasis and the tissue microenvironment. Fibroblasts are primary cells responsible for synthesizing and maintaining the ECM homeostasis and tissue integrity across diverse organs. Fibroblasts also release chemokines and cytokines in response to diseases and injuries, actively contributing to the formation of an inflammatory microenvironment within the tissue.

Fibrotic tissue is characterized by an abnormal accumulation of collagen, which displaces the typical tissue architecture and disrupts the normal function of organs. Fibroblasts, responsible for synthesizing and depositing collagen, play a central role in the formation of scar tissue upon diseases and injury. The primary biological function of fibroblasts involves synthesizing ECM proteins and maintaining ECM homeostasis (Figure 1).

The ECM serves as a foundational scaffold for cellular adhesion and tissue organization. It undergoes dynamic alterations in composition and physical characteristics during tissue healing and disease progression. Collagen, the predominant protein within the ECM, forms fibrils that contribute to tissue tensile strength. Fibroblasts play a crucial role in synthesizing all components of the ECM, including structural proteins like collagen and elastin, adhesive proteins such as fibronectin and laminin, and ground substances like glycosaminoglycans and proteoglycans. While fibroblasts are critical for repairing tissues, extended damage or persistent inflammation can interfere with the normal healing controls. Such disturbances may activate fibroblasts, leading to the development of fibrosis.

Collagen maturation is regulated by the enzyme lysyl oxidase (Lox), synthesized by fibroblasts. Lox facilitates the cross-linking of collagen fibers, thereby enhancing the stability and strength of the collagen network. Abnormalities in Lox activity have been associated with various pathological conditions, including fibrosis [10]. Lox is frequently upregulated, resulting in increased cross-linking of collagen fibers and enhanced stiffness of the ECM. This increased stiffness fosters a microenvironment conducive to fibroblast activation and augmented collagen deposition, perpetuating a fibrotic cycle. Blocking fibroblast-mediated activity of Lox to hinder collagen cross-linking can counteract both fibrosis progression and tumor metastasis [11,12,13]. Using inhibitors of collagen cross-linking shows better promise as it potentially enables endogenous proteolytic enzymes to reach previously concealed degradation sites on collagen molecules.

The regulation of collagen turnover (catabolism) involves numerous secreted extracellular proteases. Fibroblasts are a key cell type in this process by producing both enzymes that break down ECM, such as metalloproteinases (MMPs) [14,15,16], and tissue inhibitors of metalloproteinases (TIMPs) [17]. Collagen degradation is essential for preventing the formation of permanent scar tissue upon injury [18]. Under physiological conditions, collagen is continuously degraded and maintains a homeostasis where production and degradation rates are precisely balanced to prevent fibrosis development. However, in the context of fibrotic scarring, the degradation of collagen fails to keep pace with its production, resulting in the accumulation of fibrillar collagen [19]. Despite this, the biological functions of the MMPs in fibrotic scarring are complex. For instance, numerous MMPs, such as MMP-1, 2, 3, 7, 9, and 14, are elevated in the lungs of human patients with fibrosis [20]. In contrast, several experimental models of fibrosis in mice deficient in proteolytic enzymes show that MMP knockout mice, including those deficient in MMP-3, MMP-7, and MMP-8, exhibit protection against bleomycin-induced lung fibrosis [18,21,22]. These animal models underscore the complexity of the biological roles of MMPs in fibrotic scarring, indicating the need for further investigation.

Fibroblasts release growth factors, such as transforming growth factor-beta (TGF-β), a key cytokine in fibrosis, by promoting ECM synthesis and myofibroblast differentiation [23,24,25]. Fibroblasts interact with epithelial cells through paracrine signaling, influencing cell behavior and contributing to tissue homeostasis or fibrosis [9,26]. Fibroblasts can secrete pro-inflammatory cytokines and chemokines, contributing to the recruitment and activation of immune cells [27]. They interact with immune cells, such as macrophages and lymphocytes, through various signaling pathways, amplifying the inflammatory response and perpetuating fibrotic processes [28]. This inflammatory microenvironment further stimulates fibroblast activation and ECM deposition. Activated fibroblasts undergo differentiation into myofibroblasts, acquiring contractile properties like smooth muscle cells. Myofibroblasts are rarely present in healthy human tissues, but they become significantly upregulated following injury and play an essential role in tissue fibrosis [29].

Understanding the multifaceted roles of fibroblasts in tissue fibrosis is critical for developing targeted therapeutic strategies to mitigate or reverse fibrotic processes. Targeting fibroblast activation, ECM production, and the underlying signaling pathways represents a promising avenue for anti-fibrotic interventions.

## 3. Yap/Taz Signaling Pathway

Yap and Taz serve as effector components in the Hippo signaling pathway, which is a highly conserved pathway that controls cell proliferation, differentiation, and organ size regulation [30]. The pathway is triggered by diverse upstream signals such as cell density and polarity. Activation of the Hippo pathway results in the phosphorylation of Yap and Taz by the kinase complex comprising mammalian sterile 20-like kinases 1 and 2 (Mst1/2) and large tumor suppressor kinases 1 and 2 (Lats1/2).

Phosphorylation of Yap/Taz triggers their retention in the cytoplasm, followed by degradation, which inhibits their nuclear translocation. Conversely, when the Hippo pathway is inactive, Yap and Taz undergo dephosphorylation and translocate into the nucleus. Inside the nucleus, Yap and Taz function as transcriptional co-activators, engaging with various transcription factors like TEA domain family members (TEADs) to modulate the expression of target genes. Additionally, mechanical cues such as cell shape and ECM stiffness also regulate Yap/Taz activity [31,32,33]. Mechanotransduction processes influence Yap/Taz localization and activity, linking the pathway to cell adhesion and tissue mechanics. G-protein-coupled receptors (GPCRs) and their associated signaling pathways can regulate Yap/Taz activity [7,34]. Activation of GPCRs can influence Yap/Taz through various mechanisms, including changes in intracellular calcium levels and activation of downstream effectors.

Yap/Taz regulates the expression of genes crucial for cell proliferation and apoptosis, fundamental processes vital for tissue development, regulation of organ size, and regeneration. Activation of Yap/Taz is often associated with tissue repair and regeneration processes, especially in organs with high regenerative capacity, such as the liver and intestine. Yap/Taz influence cell fate determination and differentiation by modulating the expression of lineage-specific genes. Their activity is tightly regulated to ensure proper tissue development and maintenance. Yap/Taz have been associated with the control of cellular metabolism [34]. Their activity is influenced by nutrient availability, and they can modulate the expression of genes involved in metabolism. This connection between Yap/Taz and metabolism contributes to overall cellular homeostasis.

**Figure 2:**
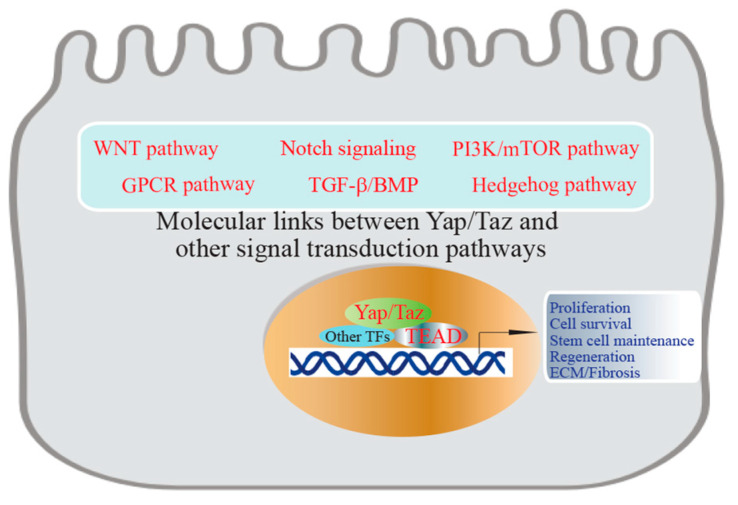
The interplay between Yap/Taz and other signaling pathways. Yap/Taz interact with diverse integrating signals. The distinctive features of Hippo signaling appear to interact with multiple integrating signals and its ability to regulate transcriptional activity through co-activator proteins that can interact with transcription factors from different pathways.

Yap/Taz signaling is associated with various diseases, including cancer, where increased Yap/Taz activity can promote uncontrolled cell growth [7]. Targeting the Yap/Taz signaling is being explored as a potential therapeutic strategy for cancer treatment. Figure 2 illustrates a wealth of interconnections between Yap/Taz and other signaling pathways (Figure 2) [7,35,36].

Noteworthy interactions include those with Wnt, Notch, and TGF-β, through which Yap/Taz coordinate cellular responses. While crosstalk among signaling pathways is common, the distinctive feature of Yap/Taz signaling lies in its integration with other pathways. This integration operates at various levels, with numerous instances of upstream-to-downstream interaction documented, many of which play crucial roles in regulating growth functions associated with the Yap/Taz pathway. Moreover, in certain instances, such as with the Wnt and TGF-β pathways, even more intricate integration occurs, facilitated by interactions among their respective transcription factors.

## 4. Yap/Taz Regulate Collagen Homeostasis

Our current understanding of the role and regulation of Yap/Taz signaling in maintaining ECM homeostasis is limited. Recently, we reported that Yap/Taz protein levels were remarkably reduced in aged human skin dermal fibroblasts in vivo [37,38] due to age-related fragmentation of collagen fibrils and subsequent reduced fibroblast stretching and mechanical forces [14,15,17,39]. Interestingly, age-related impairment of Yap/Taz signaling contributes to reduced production and increased fragmentation of collagen, which are prominent features of aged human skin dermis (dermal aging) [39]. The declined Yap/Taz activity among dermal fibroblasts due to aging has been observed in an animal model of dermal aging [40]. The decline of Yap/Taz, linked to age-related mechanical defects in the dermal ECM, emerges as a pivotal factor in dermal aging. Analysis using single-cell RNA-seq unveiled a notable decrease in the Yap/Taz signature gene set as the primary hallmark in aged mouse dermal fibroblasts. Furthermore, targeted elimination of Yap/Taz in dermal fibroblasts in young mice accelerated skin aging. Significantly, reinstating Yap/Taz activity in dermal fibroblasts by employing the constitutively active YAPS127A effectively reversed the typical aging trajectory in fibroblast-specific Yap/Taz knockout mice. These findings strongly support that reduced Yap/Taz activity in dermal fibroblasts plays a crucial role in dermal aging, underscoring the substantial significance of Yap/Taz in regulating collagen homeostasis during the skin aging process.

**Figure 3:**
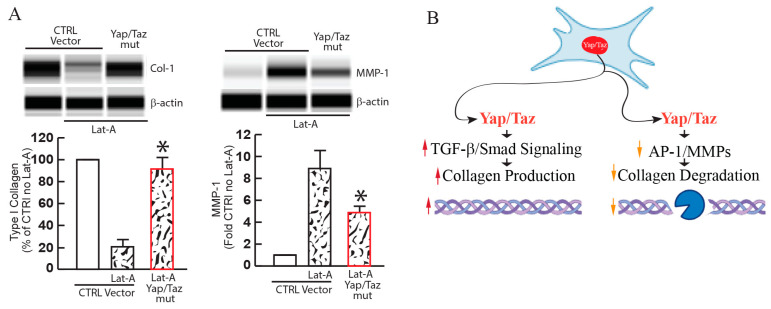
Yap/Taz regulates collagen homeostasis. (**A**) YAP/TAZ mediates aberrant collagen homeostasis in response to reduced fibroblast spreading/mechanical force. Primary adult dermal fibroblasts were transfected with the control or Yap/Taz active mutant (Yap/Taz mut) expression vectors. Two days post-transfection, the actin cytoskeleton was disrupted by Latrunculin-A (180 nM, 24 h), which reduces cell spreading/mechanical force. Type I collagen (left) and MMP-1 (right) protein levels were determined by capillary electrophoresis immunoassay and normalized to β-actin (loading control). Band intensities were quantified by Compass software (SW 5.0.1, ProteinSimple, San Francisco, CA, USA). Bands show representative digital images (mean ± SEM), N = 3. * *p* < 0.05 vs. control Lat-A. (**B**) Yap/Taz regulates collagen homeostasis. Yap/Taz upregulates collagen production by enhancing TGF-β/Smad signaling and downregulates MMP expression by inhibiting AP-1.

Latrunculin A, which diminishes cellular spreading and mechanical force by disrupting the actin cytoskeleton, causes Yap/Taz to localize in the cytosol, thereby inhibiting their transcriptional activity [31,37,38]. Latrunculin A-mediated disruption of the cytoskeleton reduces collagen and elevates MMP-1 in primary dermal fibroblasts. Importantly, the expression of active forms of Yap/Taz, which localize in the nucleus, reverses these alterations (Figure 3A). These data support the novel concept that Yap/Taz are essential for fibroblast activation and function as key regulators in collagen homeostasis by regulating collagen production and degradation. Mechanistically, Yap/Taz regulates TGF-β/Smad3 signaling by Smad7 induction via activator protein-1 (AP-1) in human skin dermal fibroblasts [41]. The TGFβ family of proteins modulates a wide array of physiological and pathological processes through Smad proteins, which serve as central regulators of TGF-β signaling [42]. The combined knockdown of Yap/Taz through siRNA impedes Smad3 phosphorylation and Smad reporter activity induced by TGF-β1, thereby inhibiting the expression of TGF-β target genes such as collagen. Diminished Yap/Taz activity initiates the upregulation of inhibitory Smad7, which subsequently blocks Smad3 phosphorylation and Smad reporter activity in response to TGF-β1 [41]. Conversely, preventing the induction of Smad7 restores Smad3 phosphorylation and Smad reporter activity in Yap/Taz-knockdown cells. Consistent with these observations, impeding Yap/Taz nuclear translocation by disrupting the cytoskeleton or limiting cell spreading also induces Smad7 and disrupts the TGF-β/Smad signaling pathway. Additionally, compromised Yap/Taz function leads to the activation of AP-1, a significant driver of Smad7 and MMP expression. As impaired Yap/Taz signaling enhances the binding of the AP-1 transcription factor to the Smad7 gene promoter, deleting the AP-1 binding site from the Smad7 promoter almost completely eliminates Smad7 promoter activity. All these pieces of evidence suggest that Yap/Taz plays an important role in regulating collagen homeostasis by controlling both TGF-β/Smad signaling and AP-1 activity (Figure 3B).

## 5. Yap/Taz Signaling in Tissue Fibrosis

Yap/Taz signaling has been demonstrated to play a role in organ fibrosis [6,7,8,43]. Yap/Taz signaling drives pathological activation of fibroblasts in pulmonary fibrosis [44] and hepatic stellate cells in liver fibrosis [45,46,47]. Additionally, Yap/Taz activation in interstitial myofibroblasts is implicated in promoting kidney fibrosis [48]. Furthermore, elevated Yap and Taz activity in non-fibroblast cell types, such as macrophages [49,50], epithelial cells [51,52,53], and hepatocytes [19,54], exacerbates the progression of fibrosis in various tissues.

**Figure 4:**
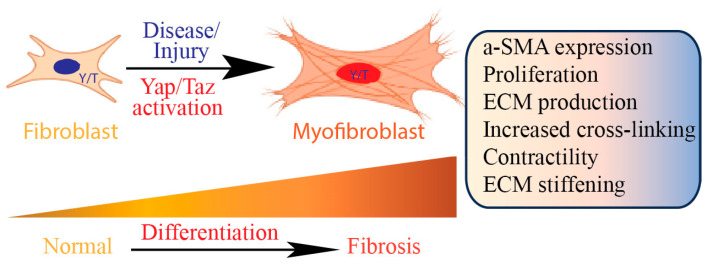
Yap/Taz mediates fibroblast differentiation into myofibroblast. Upon diseases and injuries, Yap/Taz facilitates the transformation of fibroblasts into myofibroblasts, a pivotal pathogenic process in the activation of quiescent fibroblasts leading to fibrosis.

Yap/Taz signaling activates fibroblasts and upregulates the expression of genes associated with the production of the ECM, such as collagen, fibronectin, and other matrix proteins. Persistent activation of Yap/Taz in fibroblasts stimulates fibroblast differentiation to myofibroblasts [55]. Myofibroblasts are pivotal in triggering the activation of quiescent fibroblasts, initiating fibrosis, which is a central pathological mechanism in tissue fibrosis (Figure 4). Myofibroblasts play an active role in tissue contraction and remodeling, contributing to the increased tissue stiffness observed in fibrosis. The distinctive feature of myofibroblasts lies in their expression of contractile proteins like α-smooth muscle actin (α-SMA). This transition in phenotype serves as a prominent indicator of fibrosis and is influenced by a range of signaling pathways. Yap/Taz signaling is also implicated in the process of epithelial–mesenchymal transition [56], where epithelial cells lose their characteristics and acquire a more mesenchymal, fibroblast-like phenotype [57]. This process contributes to the pool of activated fibroblasts and enhances the fibrotic response. Yap/Taz activation in fibroblasts can stimulate cell proliferation and inhibit apoptosis, leading to the expansion of the fibroblast population in fibrotic tissues. This sustained cell survival and proliferation contribute to the persistent fibrotic response.

Angiogenesis and fibrosis are intricately linked processes that often occur simultaneously in fibrotic conditions [9]. Yap/Taz has been shown to govern endothelial cell activities such as proliferation, migration, and survival, thereby influencing processes like vascular sprouting, formation of vascular barriers, and remodeling of blood vessels [58]. Fibroblasts communicate with vascular endothelial cells, influencing angiogenesis and vascular remodeling, which are integral processes in tissue repair and fibrosis. Fibroblast Yap/Taz signaling plays an important role in secreting pro-angiogenic factors such as vascular endothelial growth factor (VEGF), fibroblast growth factor (FGF), and angiopoietin-2 [59,60,61,62]. These factors act on endothelial cells, promoting their proliferation, migration, and tube formation, which are essential steps in the formation of new blood vessels. Notably, dermal fibroblasts from patients with systemic sclerosis exhibit heightened VEGF expression in response to autocrine TGF-β signaling [63]. This upregulated VEGF may contribute to vascular remodeling, thus fostering fibroblast activation and supporting fibrosis [63]. In addition, ECM proteins are essential for the migration of endothelial cells to form tubes within the ECM connective tissue [64]. Fibroblast Yap/Taz signaling is required for the production of ECM proteins necessary for endothelial cell-mediated lumen formation and angiogenesis [61,65].

Yap/Taz signaling is significantly involved in the formation of fibrotic scarring in the skin, a phenomenon frequently seen following various types of damage such as injury, burns, infections, and persistent inflammation [1,6,66]. This process can result in the formation of raised, thickened areas of scar tissue, such as keloids or hypertrophic scars. Conditions like systemic sclerosis, systemic lupus erythematosus, and severe burns can also lead to fibrotic scarring. Studies have indicated that Yap and Taz are activated in response to skin injury and play a role in regulating the behavior of various cell types involved in wound healing, including fibroblasts [67]. Activation of Yap/Taz signaling in keloid fibroblasts has been associated with increased collagen production, fibroblast activation, and myofibroblast differentiation, all of which are key processes in fibrotic scarring. Furthermore, dysregulated Yap/Taz signaling has been implicated in the pathogenesis of fibrotic skin diseases such as systemic sclerosis and hypertrophic scarring [66]. Fibrotic scar formation in skin is a complex process, as fibroblasts exhibit heterogeneity in skin dermis. For example, in keloid scar formation, fibroblasts can be classified into four distinct subgroups, i.e., secretory-papillary, secretory-reticular, mesenchymal, and pro-inflammatory [68]. Notably, the mesenchymal fibroblast subset shows a significant increase in keloid tissue compared to normal scar tissue. Functional investigations highlight the critical role of mesenchymal fibroblasts in the overexpression of collagen during keloid formation. Furthermore, an increased presence of mesenchymal fibroblasts is also noted in other fibrotic skin conditions, such as systemic sclerosis, suggesting a widespread mechanism contributing to skin fibrosis. These discoveries will help better understand the pathogenesis of skin fibrosis and offer potential therapeutic targets for fibrotic diseases.

CCN2/CTGF belongs to the matricellular protein family called CCN [69,70,71]. While CCN2/CTGF is widely acknowledged as an established downstream target of Yap/Taz [72,73], it often serves merely as an indicator of Yap/Taz activity rather than functioning as downstream effectors of Yap/Taz signaling. CCN2/CTGF is significantly elevated in fibrotic tissues and considered both a mediator and a biomarker of tissue fibrosis [74,75]. However, investigation of the role of CCN2/CTGF in mediating Yap/Taz signaling in tissue fibrosis remains relatively limited. In aged human skin dermal fibroblasts, the levels of CCN2 are notably lower when compared to those in young skin dermal fibroblasts [37,76]. This reduction contributes to the process of dermal aging by inhibiting collagen production [37,76]. Our recent investigation uncovered that the diminished expression of CCN2 in aged human skin dermal fibroblasts is a consequence of the age-related deterioration of Yap/Taz signaling [37]. The restoration of impaired Yap/Taz signaling associated with aging successfully reversed the reduction in CCN2 expression in human skin fibroblasts. These findings indicate that Yap/Taz plays a pivotal role in the age-related decline of CCN2/CTGF, implicating their involvement in dermal aging via the downregulation of CCN2/CTGF. This underscores the significance of Yap/Taz signaling in maintaining skin homeostasis and suggests potential therapeutic targets for addressing age-related skin deterioration. This suggests that Yap/Taz is responsible for the age-related decrease in CCN2/CTGF and contributes to dermal aging through the downregulation of CCN2/CTGF. Exploring the potential mediation of organ fibrosis by Yap/Taz through CCN2/CTGF presents an intriguing avenue of investigation. Delving deeper into the interplay between CCN2/CTGF and Yap/Taz in the context of organ fibrosis could yield valuable insights into the underlying mechanisms of organ fibrosis.

Yap/Taz signaling interacts with other signaling pathways involved in fibrosis, such as TGF-β and Wnt signaling [7]. The crosstalk between these pathways amplifies the fibrotic response and creates a complex network of regulatory mechanisms. As such, dysregulation or sustained activation of Yap/Taz can contribute to organ fibrosis by promoting fibroblast activation, myofibroblast differentiation, and excessive ECM production.

## 6. Yap/Taz Signaling as a Mechanical Driver of Scar Formation

Mechanical forces regulate the function of Yap/Taz [31,44]. As fibroblasts are highly mechanosensitive, recent evidence suggests that fibrotic collagen itself is essential in promoting fibrogenic cell activation that drives progressive fibrosis by impacting the mechanical environment experienced by cells within the wound scar [8,77,78]. The dynamic shifts within the mechanical landscape of the ECM wield substantial impacts on the signaling pathways and activation state of mesenchymal cells [79,80,81]. Interfering with mechanical signaling in an animal model of fibrosis has demonstrated the prospect of targeting cellular activation influenced by the mechanical environment of the matrix [82]. Nevertheless, a significant challenge lies in our comprehension of how cells transduce matrix mechanical properties and translate them into cellular signaling, which remains a significant obstacle in targeting the mechanobiological aspect of fibrosis. Pathological fibrosis is propelled by a cyclic process wherein the fibrotic ECM serves as both an instigator and outcome of fibroblast activation. This interplay forms a feedback loop, perpetuating the fibrotic condition. However, the molecular mechanisms underlying this process remain poorly understood. The stiffening of the ECM enhances the activation of Yap/Taz [7]. In various organs, including the liver [83,84], kidney [85], lung [44], and skin [86], fibroblasts exhibit nuclear Yap/Taz activation, leading to the promotion of fibrotic cellular characteristics, such as increased potential for myofibroblast differentiation and matrix remodeling.

**Figure 5:**
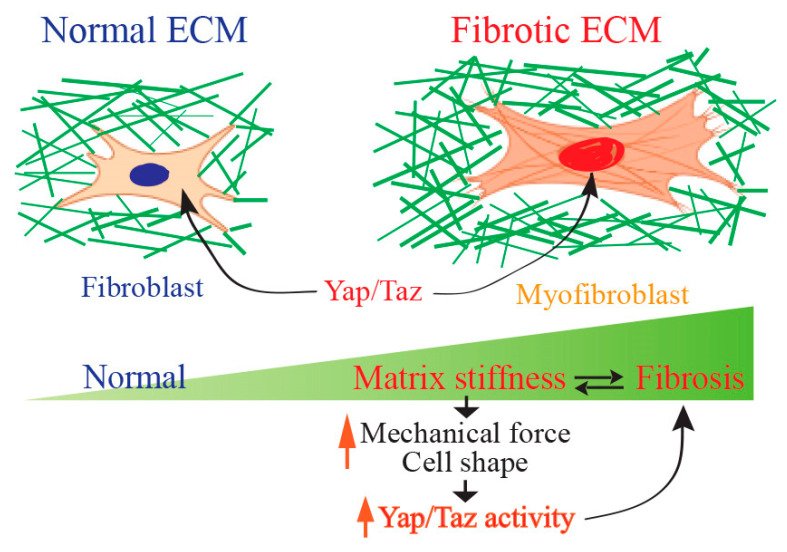
Yap/Taz signaling as a mechanical driver of tissue fibrosis. Yap/Taz signaling functions as a pivotal force in driving fibrotic matrix formation by orchestrating the activation of fibroblasts and synthesis of ECM in response to changes in the rigidity of the fibrotic matrix.

Yap/Taz serve as coordinators in regulating fibroblast activation and matrix synthesis in response to changes in matrix stiffness [37,44] (Figure 5).

In cell culture, Yap/Taz become active and produce collagenous ECM on matrices that are pathologically rigid but not on those that are physiologically compliant. When Yap/Taz are knocked down in vitro, essential fibroblast functions such as collagen synthesis, contraction, and proliferation are diminished, specifically on matrices that are pathologically stiff. Immortalized fibroblasts expressing active Yap/Taz mutant proteins surpass growth restrictions on soft matrices in vitro and trigger fibrosis upon transfer to the murine lung. These findings highlight the capacity of fibroblast Yap/Taz activation to instigate a profibrotic response in vivo.

Additionally, studies have indicated that while a soft ECM hampers, a stiff ECM enhances TGF-β-induced profibrotic Smad signaling by regulating the localization of Smad2/3, a process mediated by Yap/Taz [85].

These findings collectively designate Yap/Taz as key regulators of fibroblast mechanoactivation and fibrogenic progression.

## 7. Fibroblast Yap/Taz Function as Immune Regulators to Control Fibrotic Wound Scarring

**Figure 6:**
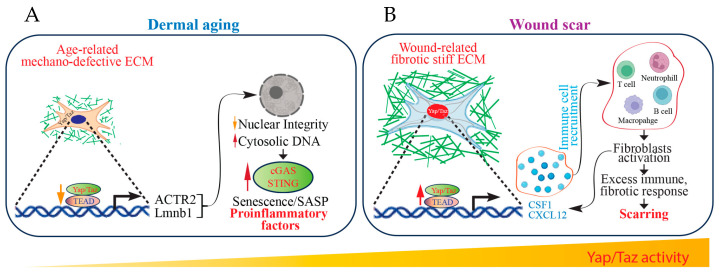
Fibroblast Yap/Taz function as immune regulators to control fibrotic wound scarring. Yap/Taz signaling serves as an immune regulator to modulate skin ECM homeostasis. (**A**) Yap/Taz signaling suppresses the activation of the proinflammatory immune pathway mediated by cGAS-STING. Disruption of this suppression, due to loss of fibroblast shape/mechanical force caused by age-related mechano-defective dermal ECM, triggers cellular senescence, thereby contributing to inflammation. (**B**) Cytokines CSF1 and CXCL12, derived from fibroblasts, act as key regulators in priming inflammation. They orchestrate the recruitment of immune cells to specific sites, thereby promoting the process of fibrotic wound scarring.

Fibroblasts were traditionally considered ‘immune neutral’ cells, and thus fibroblast-immune interactions often remain largely unexplored. Nevertheless, recent findings propose that fibroblasts have assumed a pivotal role as immune sentinel cells, initiating and regulating immune responses in the presence of pathological stimuli [27,28,87,88]. In human skin, fibroblasts are a critical source of inflammation [87,89,90], which is a significant risk factor for multiple chronic diseases in elderly individuals [91,92,93]. Fibroblasts and immune cells interact through complex signaling pathways, working collaboratively to sculpt the intricate landscape of the tissue microenvironment. This dynamic interplay significantly influences disease outcomes, underscoring the multifaceted nature of their relationship [94]. Recently, Sinha et al. reported the exciting discovery that injured reindeer antler velvet skin regenerates without a scar, while injured reindeer back skin forms a fibrotic scar [95]. Single-cell RNA sequencing and proteomics identified that fibroblasts from antler skin wounds displayed distinct inflammatory phenotypes and signals resembling developmental and regenerative characteristics like human fetal fibroblasts. In contrast, fibroblasts from the back skin of reindeer demonstrate a transcriptional program associated with pro-inflammatory responses. Particularly, fibroblast-derived cytokines, CSF1 and CXCL12, function as master mediators of inflammatory priming and direct site-specific immune cell recruitment to promote scar formation (Figure 6B). CSF1 serves as the principal growth factor essential for regulating monocyte and macrophage differentiation, survival, proliferation, and renewal [96]. CSF1 is widely expressed, predominantly originating from cells of mesenchymal lineages. CXCL12 is a fibroblast-specific protein and significantly elevated in inflammatory human skin conditions [89]. Fibroblast-derived CSF1/CXCL12 inflammatory signals mediate fibrotic wound scarring through the recruitment of immune cells. These findings suggest that the immune system, influenced by fibroblasts, participates in the transition between regeneration and fibrotic healing. This is supported by the established observation that human fetuses, known for scarless healing, possess immature immune systems [97].

Recently, the spiny mouse (Acomys species) [98] has gained attention as an exciting research organism, owing to its extraordinary ability to heal skin wounds and ear punches without scarring [99,100]. Although the exact mechanisms driving skin regeneration in Acomys are yet to be fully understood, preliminary studies comparing Acomys with Mus, a typical laboratory mouse, indicate the significance of immune cells in coordinating the scarless regeneration of skin and ear wounds in Acomys. After an injury, the Acomys wound site exhibits a down-regulation of pro-inflammatory factors and an upregulation of pro-reparative factors compared to *Mus* [101,102,103,104]. While inflammatory macrophages (M1-type) are scarce or absent during the healing process in Acomys wounds, the experimental depletion of all macrophages results in delayed closure of ear holes in Acomys [102], akin to the regenerative failures observed in axolotls [105] and mouse digit-tips [106]. Moreover, Acomys dermal fibroblasts maintain distinctive “relaxed” biophysical characteristics [107], akin to those found in aged dermal fibroblasts, which produce less collagen due to loss of cell shape and mechanical tension [39]. These traits could potentially facilitate scar-free wound healing. The regenerative potential exhibited by Acomys species is captivating, undoubtedly offering significant promise from a translational perspective.

The Yap/Taz signaling involves initiating and sustaining an inflammatory response within fibrotic tissues [88,108]. This includes the activation of immune cells and the release of pro-inflammatory cytokines, creating a microenvironment that further promotes fibrogenesis. Fibroblast Yap/Taz signaling facilitates inflammatory gene expression and modulates the skin’s inflammatory phenotype, regulating the process of fibrotic scarring in wounds.

In contrast to fibrotic scars, age-related decline of Yap/Taz activity in dermal fibroblasts has been reported in both human skin [37,38,109] and mouse skin [40]. This age-related reduction in Yap/Taz activity contributes to the emergence of an inflammation-related dermal microenvironment [37,40,90]. Mechanistically, the decline in fibroblast shape and mechanical tension, attributed to age-related mechano-defective ECM, fosters the inflammatory dermal microenvironment [37,40,90]. Notably, the Yap/Taz signaling pathway plays a pivotal role in suppressing the proinflammatory immune cGAS-STING pathway. Removal of this inhibition induces cellular senescence, ultimately leading to inflammation and age-related tissue degeneration [40] (Figure 6A). All the evidence suggests that fibroblast Yap/Taz signaling serves as an immune regulator, influencing the inflammatory characteristics of the skin to govern the processes of wound scarring and regeneration.

## 8. ECM-Immune Microenvironment and Yap/Taz in Fibrosis

While immune cells adhere to the ECM and reside within the tissue microenvironment [88], the potential consequences of these interactions often remain unexplored. ECM, constituting at least a third of tissue structures, has received limited attention in the field of immunology. Likewise, researchers in matrix biology frequently disregard the intricate regulation of complex structural matrices by the immune system. Our comprehension of the influence of ECM structures on immune cell localization and function is only just beginning to emerge. Exploring the impact of matrix integrity and mechanical properties (such as stiffness and force) on immune cell behavior, including movement and positioning, within fibrotic tissues proves to be an intriguing field of research.

**Figure 7:**
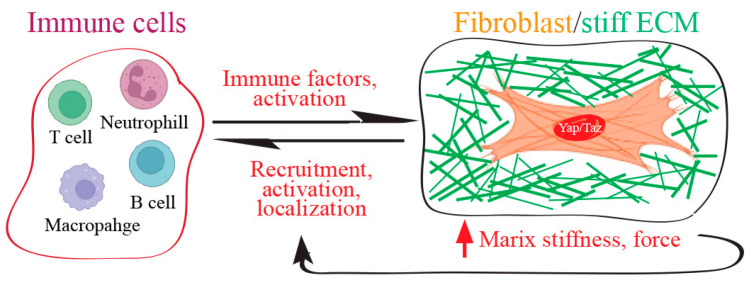
Yap/Taz is involved in ECM-immune communication in fibrosis. In the context of tissue fibrosis, there is a collaborative effort between immune cells and fibroblasts. They engage in a dynamic exchange of signals, both soluble factors and physical interactions, enabling them to adapt and integrate each other’s functions. While immune factors have the capability to activate fibroblasts, biophysical and mechanical cues mediated by Yap/Taz, such as matrix stiffness and mechanical forces, play a pivotal role in shaping the behavior of immune cells during the process of fibrotic scarring.

The immune system and fibroblasts collaborate during fibrosis in response to tissue wound repair. Both cell types are integral to these processes, communicating through soluble and physical cues and adopting functions from each other [94]. The biophysical/mechanical cues, including matrix stiffness and mechanical forces, can influence immune cell behavior [110] (Figure 7). The increased stiffness of the ECM in fibrotic tissues has been observed to induce inflammatory activation of macrophages through activation of Yap [111]. In contrast, macrophage adhesion to soft hydrogels, as opposed to stiff materials, results in diminished inflammation and is associated with hindered Yap activation. The depletion of Yap inhibits macrophage inflammation, while the overexpression of active Yap enhances inflammation. Soft materials also lead to a reduction in the expression of inflammatory markers and Yap in adjacent macrophages compared to stiff materials. Taken together, these observations underscore the significance of Yap as a central molecule in governing inflammation and sensing stiffness in macrophages, potentially offering wide-ranging implications for the modulation of macrophages in both physiological and pathological conditions.

The interaction between the ECM and immune cells is essential, as the structural and mechanical properties of the ECM significantly influence the movement, viability, and activity of immune cells [88]. Conversely, the immune system actively oversees and regulates the matrix integrity following injuries. When this ECM-immune system partnership is disrupted, it becomes a significant factor in the development of various diseases. Investigating the intricate connections between ECM biology and immune cells presents an opportunity to advance scarless wounds and promote the maintenance of healthy organs.

## 9. Future Perspectives and Challenges

Future investigations are poised to uncover the precise regulatory mechanisms of Yap/Taz in tissue fibrosis. Such studies may aim to modulate Yap/Taz activity and/or disrupt downstream signaling pathways, offering promising avenues for the treatment of fibrotic diseases affecting diverse organs.

The interplay between fibroblasts, particularly those generating inflammatory signals, and the immune system is pivotal in mediating the shift from regeneration to fibrotic healing. A fundamental question emerges: Could blocking inflammatory signals originating from fibroblasts potentially prevent wound scarring and promote regeneration? To address this critical question, there is a strong need for an appropriate animal model featuring a targeted knockout of Yap/Taz specifically in fibroblasts.

Pathological fibrosis is driven by a positive feedback loop, where fibrotic collagen serves as both a trigger and an outcome of fibroblast activation. Presently, much of the scientific endeavor in fibrosis concentrates on cell-autonomous factors and pathways that regulate increased collagen production rather than exploring fibrotic tissue degradation as a therapeutic strategy. An important investigation entails examining the fibrotic pathological matrix as a possible path for therapeutic intervention. Can mitigating mechanical properties in scar tissue, like tissue stiffness or mechanical tension, hinder Yap/Taz activity, ultimately leading to reduced wound scarring and the encouragement of regeneration? The strategy of partially breaking down collagen fibrils and/or reducing cross-linking of the collagen fibrils in scar tissue leads to decreased scar stiffness/mechanical tension; compromised Yap/Taz signaling is caused by diminished fibroblast spreading/mechanical force by disrupting cell-collagen interaction.

A significant challenge in the field of fibrosis lies in the intricate relationship between ECM and immune cells, coupled with our insufficient understanding of the communication between these two entities. Several cytokines secreted by immune cells exhibit profibrotic activity. Among these are interleukin (IL)-4, IL-5, IL-13, IL-33, IL-17A, interferon-γ, and tumor necrosis factor (TNF) [94]. These cytokines primarily exert their profibrotic activity through interactions with fibroblasts. However, whether and how these cytokines influence Yap/Taz signaling in fibroblasts is largely unexplored, despite the possibility that Yap/Taz may be involved. For instance, in HEK293 (human embryonic kidney cells), Yap/Taz serves as a mediator for TNF-induced proinflammatory and profibrotic gene expression [112]. Exploring the intricate relationship between ECM biology and immune cells offers a promising avenue for developing innovative strategies to enhance wound healing while minimizing scarring. To achieve this objective, it is imperative for immunologists and matrix biologists to collaborate closely.

Postsurgical adhesion presents a significant challenge in surgeries, impacting patient outcomes and overall quality of life. Numerous studies have reported the involvement of various signaling pathways in adhesion formation [113]. However, due to the interdisciplinary nature of this research, our understanding of adhesion mechanisms remains limited. Mechanism-based strategies for preventing or minimizing adhesion formation are imperative. The influence of the Yap/Taz pathway on adhesion formation is still being investigated. Dysregulation of the Yap/Taz pathway is known to contribute to excessive scar tissue formation, underscoring the need for future research to elucidate its role in the adhesion process.

Achieving wound healing without scarring remains a challenge. Understanding the roles of fibroblast Yap/Taz signaling in organ fibrosis is important for developing targeted therapeutic strategies to mitigate or reverse the fibrotic process.
